# Caveolin-1 Associated Adenovirus Entry into Human Corneal Cells

**DOI:** 10.1371/journal.pone.0077462

**Published:** 2013-10-11

**Authors:** Mohammad A. Yousuf, Xiaohong Zhou, Santanu Mukherjee, Ashish V. Chintakuntlawar, Jeong Yoon Lee, Mirja Ramke, James Chodosh, Jaya Rajaiya

**Affiliations:** Department of Ophthalmology, Harvard Medical School, Massachusetts Eye and Ear Infirmary, Boston, Massachusetts, United States of America; UC Berkeley, United States of America

## Abstract

The cellular entry of viruses represents a critical area of study, not only for viral tropism, but also because viral entry dictates the nature of the immune response elicited upon infection. Epidemic keratoconjunctivitis (EKC), caused by viruses within human adenovirus species D (HAdV-D), is a severe, ocular surface infection associated with corneal inflammation. Clathrin-mediated endocytosis has previously been shown to play a critical role in entry of other HAdV species into many host cell types. However, HAdV-D endocytosis into corneal cells has not been extensively studied. Herein, we show an essential role for cholesterol rich, lipid raft microdomains and caveolin-1, in the entry of HAdV-D37 into primary human corneal fibroblasts. Cholesterol depletion using methyl-β-cyclodextrin (MβCD) profoundly reduced viral infection. When replenished with soluble cholesterol, the effect of MβCD was reversed, allowing productive viral infection. HAdV-D37 DNA was identified in caveolin-1 rich endosomal fractions after infection. Src kinase activity was also increased in caveolin-1 rich endosomal fractions after infection, and Src phosphorylation and CXCL1 induction were both decreased in caveolin-1-/- mice corneas compared to wild type mice. siRNA knock down of caveolin-1 in corneal cells reduced chemokine induction upon viral infection, and caveolin-1-/- mouse corneas showed reduced cellular entry of HAdV-D37. As a control, HAdV-C2, a non-corneal pathogen, appeared to utilize the caveolar pathway for entry into A549 cells, but failed to infect corneal cells entirely, indicating virus and cell specific tropism. Immuno-electron microscopy confirmed the presence of caveolin-1 in HAdV-D37-containing vesicles during the earliest stages of viral entry. Collectively, these experiments indicate for the first time that HAdV-D37 uses a lipid raft mediated caveolin-1 associated pathway for entry into corneal cells, and connects the processes of viral entry with downstream proinflammatory cell signaling.

## Introduction

Human adenoviruses (HAdVs) cause a broad range of infections at mucosal sites [1]. Additionally, HAdV vectors are widely used for the study of cellular processes, and are chosen as gene and vaccine delivery vehicles for clinical use. A shortcoming of HAdVs in therapy for human diseases is their intrinsic tendency, as with infection by naturally occurring HAdVs, to induce innate immune responses and inflammation [2,3]. One potential mechanism for HAdV induced inflammation can be found in the process of viral entry, in which cellular components involved in viral internalization directly induce subsequent inflammatory gene expression by infected cells [4–7]. Therefore, to understand basic mechanisms of HAdV induced inflammation, and to mitigate complications of adenoviral gene therapy, it is important to understand modes of HAdV entry into various cell types. 

Endocytosis is a multi-step process that involves internalization of macromolecules and particles into specific transport vesicles derived from a cell’s plasma membrane. Clathrin mediated endocytosis is a well studied endocytic pathway used by both enveloped and non-enveloped viruses [8].The cell surface is abundantly endowed with other components that allow detection and response to external stimuli. Lipid rafts represent one of the major constituents of the cell membrane, contain a heterogeneous population of proteins, and act as centers for downstream cell signaling. Caveolae are a special type of lipid raft that by electron microscopy appear as uncoated, flask-shaped invaginations of the plasma membrane, and are responsible for uptake and efflux of cholesterol esters. Caveolar, lipid raft mediated endocytosis plays an important role in cell adhesion and growth [9,10]. Caveolins are the major integral membrane protein of caveolae [11] and include three types with a similar molecular weight of 22 kDa [12,13]. Caveolin-1 and -2 are found in most cell types, while caveolin-3 is present only in muscle cells. Knockout of the gene for caveolin-1 in mice also reduces caveolin-2 expression, and leads to loss of morphologically defined caveolae [14]. Caveolae-like structures were identified as possible pathways of viral entry for SV40 in 1989 [15], however, SV40 entry via caveolae was recently disputed [16]. In addition to SV40, caveolae and lipid rafts have been implicated in the entry of filovirus [17], human enterovirus [18], echovirus [19] and human immunodeficiency virus [20,21], among others [22–26]. Importantly, the specific means of host cell entry by viruses appears to be dependent on the type of cell and its state of activation, and may be redundant [27,28].

Signaling molecules are an integral part of the host immune response to viral infection, and recent studies have shown a role for cell signaling in viral entry [29–32] and viral replication [33]. For adenoviruses, viral attachment to specific integrins activates PI3K which in turn activates the small GTPases, Rac and Cdc42 [34] . Inhibition of cell signaling can drastically reduce virus infection [35,36]. SV40 entry is regulated by at least five different kinases [37]. Virus attachment to cells can induce lipid raft formation by clustering of specific surface proteins and lipids. Interestingly, many viruses bind glycophosphatidylinositol (GPI)-anchored proteins and gangliosides, associated with the outer leaflet of the plasma membrane, and this may lead directly to activation of cellular kinases [30], adding to increasing evidence that virus binding to lipid rafts specifically initiates intracellular signaling. 

For the entry of adenoviruses, existing evidence suggests entry begins with a requisite binding of the adenovirus capsid fiber knob to a cellular receptor such as the coxsackie adenovirus receptor (CAR) [38], CD46 [39,40], or in particular for viruses causing epidemic keratoconjunctivitis, GD1a glycan [41]. Initial binding is followed by a secondary interaction between the arginine-glycine-aspartic acid (RGD) motif in the viral capsid penton base and cellular integrins α_v_β_3_, α_v_β_5,_ and α_V_β_1_ [42–45] This secondary interaction is thought to induce a host cell signaling cascade resulting in clathrin mediated endocytosis [46–48], and possibly, activation of the rab5 dependent classical endosomal pathway [49]. Herein, we show that HAdV-D37, an etiologic agent of epidemic keratoconjunctivitis, enters primary human corneal fibroblasts predominantly via lipid rafts and caveolae, suggesting both redundancy and cell specificity in mechanisms of adenoviral entry. 

## Results

### Cell and virus specific infection

**Figure 1:**
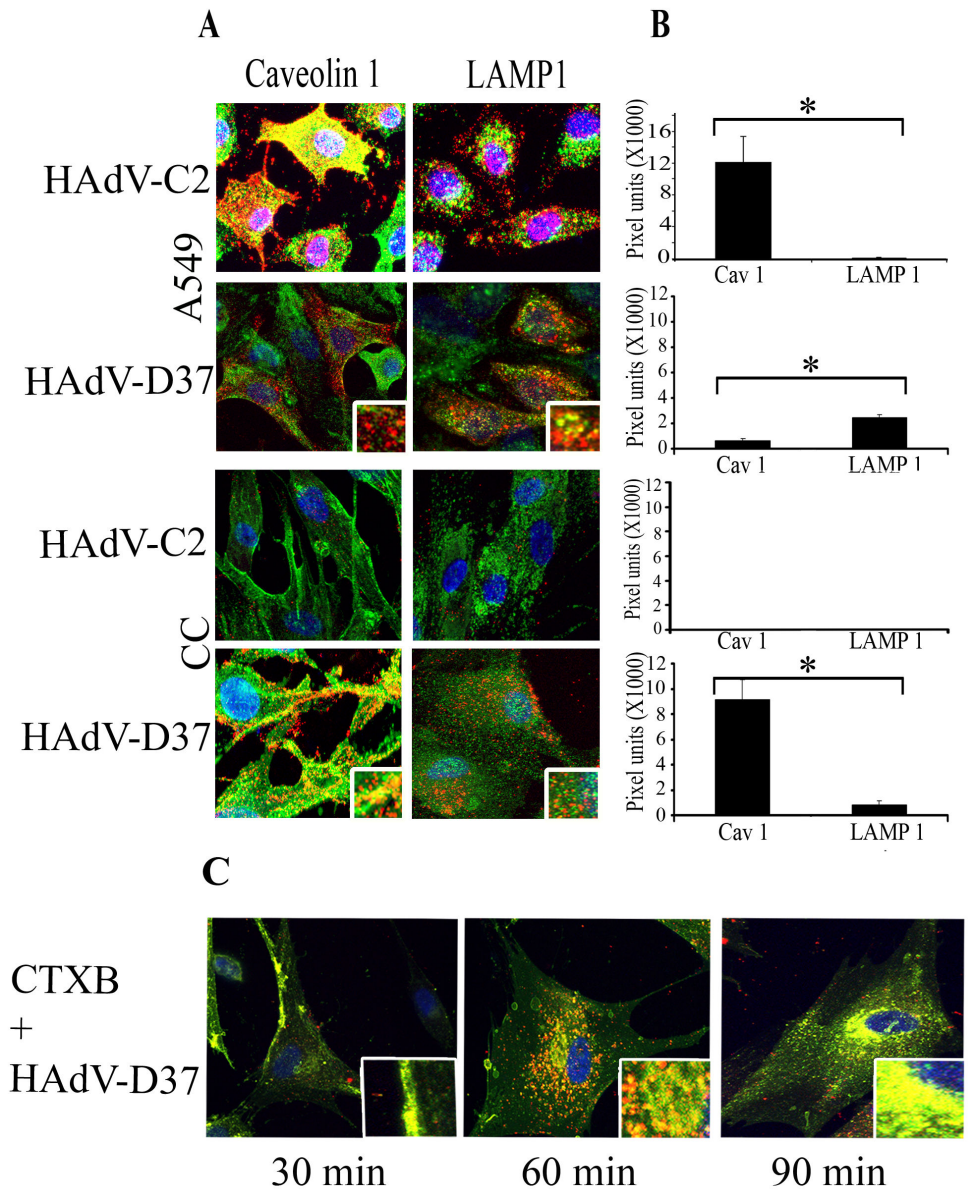
HAdV-37 uses caveolin-1 to enter human corneal cells. **A**. Human corneal and A549 cells grown on slide chambers were infected with Cy3-labeled HAdV-D37 (red). Cells were then stained with caveolin-1 or LAMP1 antibodies as indicated followed by alexa-fluor488 (green) secondary antibodies. Original magnification: 63X. Insets represent similarly magnified squares from each photomicrograph. **B**. Three random frames from different experiments were chosen in masked fashion from each experiment (n=3) to quantify co-localization using Amira 5.2.2. In A549 cells, HAdV-C2 co-localized predominantly with caveolin-1, while HAdV-D37 co-localized with LAMP1 (*p=.02 and *p=.0025, respectively). In corneal cells (CC), Cy3-labeled HAdV-D37 co-localized predominantly with caveolin-1 (*p=.0068), and HAdV-C2 was not seen. Students *t* test was used for all comparisons. **C**. Human corneal cells were grown in chamber slides, Cy3-labelled HAdV-D37 and 488-Cholera Toxin B (CTXB) were added to the cells on ice and then warmed to 37°C, and incubated for 30, 60, and 90 min, prior confocal microscopy. HAdV-D37 (red) co-localized with CTXB (green) at all time points. At 30 min after warming, HAdV-D37 and CTXB co-localization was observed at the cell membrane, at 60 min in the cytoplasm, and at 90 min at the perinuclear region.

To test whether viral entry is cell and/or virus specific as previously shown for HAdV-C [50], we directly compared pathways of infection in human corneal fibroblasts and A549 cells using HAdV-D37 and HAdV-C2. Individual confocal image slices were loaded to measure co-localization pixels (Amira 5.2.2) in cells (green: caveolin-1 or LAMP1, a late endosomal marker associated with conventional endosomes [51], red: Cy3-labeled HAdV-D37). In human corneal cells, Cy3-labeled HAdV-D37 co-localized to a greater degree with caveolin-1 than with LAMP1 (p=.0068), while in A549 cells HAdV-37 predominantly co-localized with LAMP1 (p=.0025), indicating an entry pathway other than caveolae (Figures 1A and B). HAdV-C2, not known to infect the cornea, failed to infect corneal cells in culture, and in A549 cells co-localized predominantly with caveolin-1 (p=.02). (Figures 1A and B). Cholera toxin B subunit (CTXB) has been used to track and identify lipid rafts. To further examine the role of lipid raft microdomains in HAdV-D37 entry, we treated corneal cells with both Cy3-labeled virus and 488-CTXB, and tracked their cellular localization over time (Figure 1C). Co-localization of virus and CTXB was observed in the cell membrane at 30 min, cytoplasm at 60 min, and perinuclear region at 90 min, supporting a role for caveolin-1 and lipid rafts in HAdV-D37 entry into corneal cells.

### Cholesterol Dependent HAdV-D37 Cell Entry

**Figure 2:**
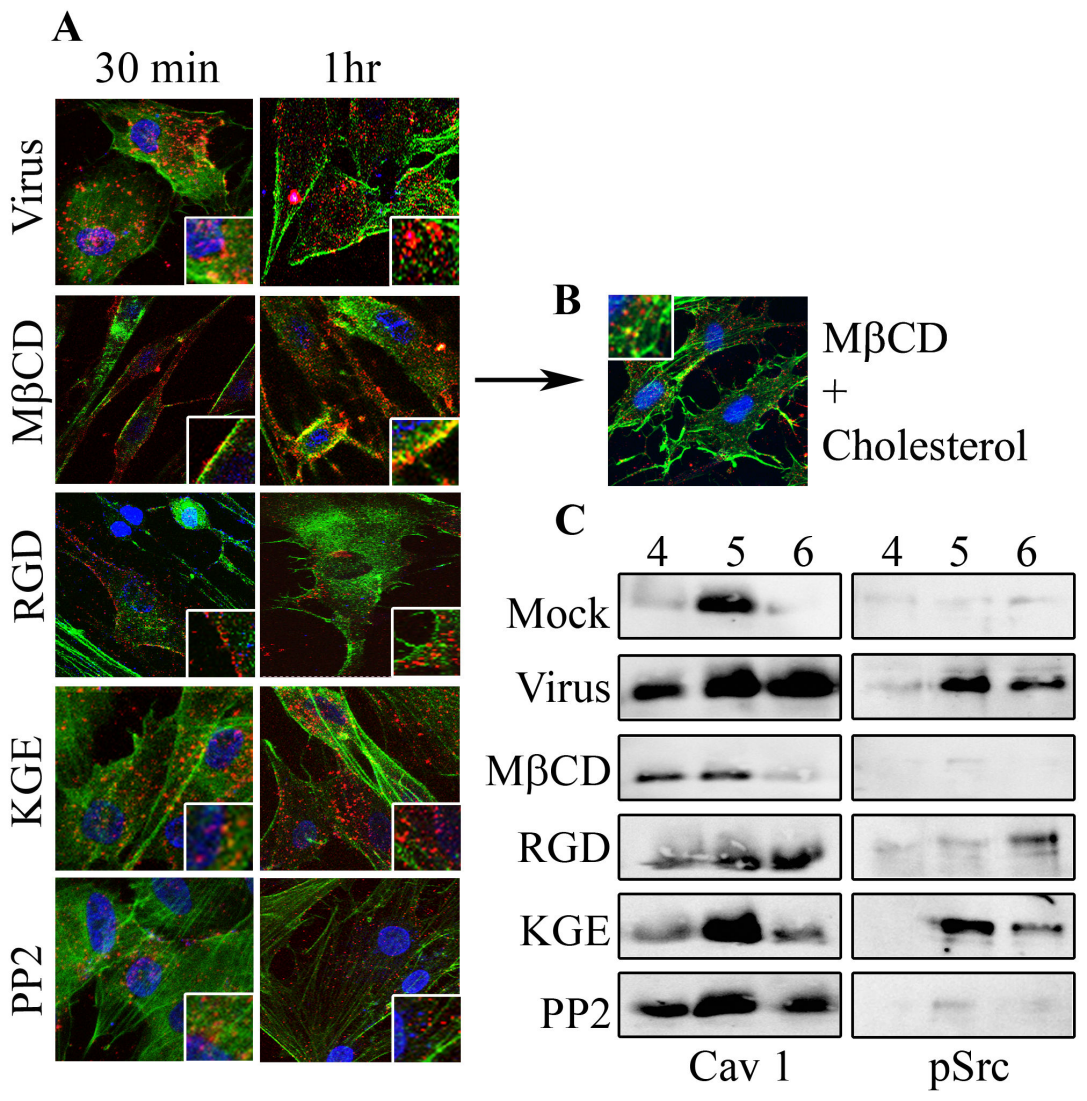
Cholesterol depletion impedes viral entry. **A**. Human corneal cells grown on slide chambers were pretreated with MβCD (5 mM), RGD-containing 15-mer (50 μM), a KGE-containing 15-mer otherwise identical to the RGD 15-mer (50 μM), or the Src kinase inhibitor PP2 (10 μM), and then infected with Cy3-labeled HAdV-D37 (red). MβCD pretreatment prevented virus from entering the cells both at 30 min and 1 hr post infection as compared to no pretreatment. RGD and PP2 reduced entry as compared to KGE treated or untreated cells. Co-staining with alexa-fluor488 phalloidin (green). Original magnification: 63X. Insets represent similarly magnified squares from each photomicrograph. **B**. Cholesterol was added to MβCD pretreated cells for one hr and then infected with Cy3-labeled HAdV-D37. Confocal microscopy revealed entry of viruses into the corneal cells. **C**. Detergent free lipid raft preparations in HAdV-D37 infected corneal cells, pretreated with MβCD, RGD 15-mer, KGE 15-mer, or PP2, and lysed at 1 hr post infection, followed by immunoblotting for caveolin-1 or phosphorylated Src (pSrc). Blots show increased caveolin-1 and pSrc upon viral infection, but reduced expression with cholesterol depletion or blocking of integrin aggregation (MβCD and RGD treatment, respectively). PP2 treatment did not effect caveolin-1 levels in infected cells, but did reduce pSrc.

In order to directly determine a role for cholesterol-laden lipid rafts in HAdV-D37 entry, primary corneal cells were pretreated with MβCD (5 mM) to deplete cholesterol prior to infection with Cy3-labeled HAdV-D37. Earlier studies showed an essential role for both integrins and Src kinase in HAdV entry [31,35,45,46,52]. Therefore, control pretreatments also included an RGD-containing 15-mer (50 μM) designed to match the amino acid sequence in the HAdV-D37 penton base (DAVP(RGD)NYASAAEA) [53,54], in order to block integrin aggregation, and its KGE-containing 15-mer control (DAVP(KGE)NYASAAEA), otherwise identical to the RGD 15-mer. The chemical PP2 (10 μM) was used to inhibit Src kinase activity. At both 30 min and 1 hr post infection, viral entry appeared nearly completely blocked by pretreatment with either MβCD or RGD, and partially by PP2, but not by KGE (Figure 2A). Replenishment of cholesterol (0.4mg/ml in 0.2mM MβCD) for 1 hr appeared to restore both normal cell morphology and viral entry, as assessed by confocal microscopy (Figure 2B). Western blot analysis of detergent free lipid raft preparations at 1 hr post infection revealed increased caveolin-1 in lipid rafts from virus infected cells as judged collectively in fractions 4, 5 and 6 (Figure 2C). Caveolin-1 was seen at much lower levels and only in the 5^th^ fraction of mock infected cells. In cells that were MβCD treated prior to virus infection, caveolin-1 was reduced. We also observed increased phospho-Src (pSrc) in virus infected cell fractions 5 and 6. Reductions in both caveolin-1 and pSrc were observed in RGD pretreated cells, whereas PP2 pretreatment reduced only pSrc and not caveolin-1 (Figure 2C). These results suggest a relationship between caveolin-1 and Src in HAdV-D37 entry.

### HAdV-D37 enters corneal cells via caveosomes

**Figure 3:**
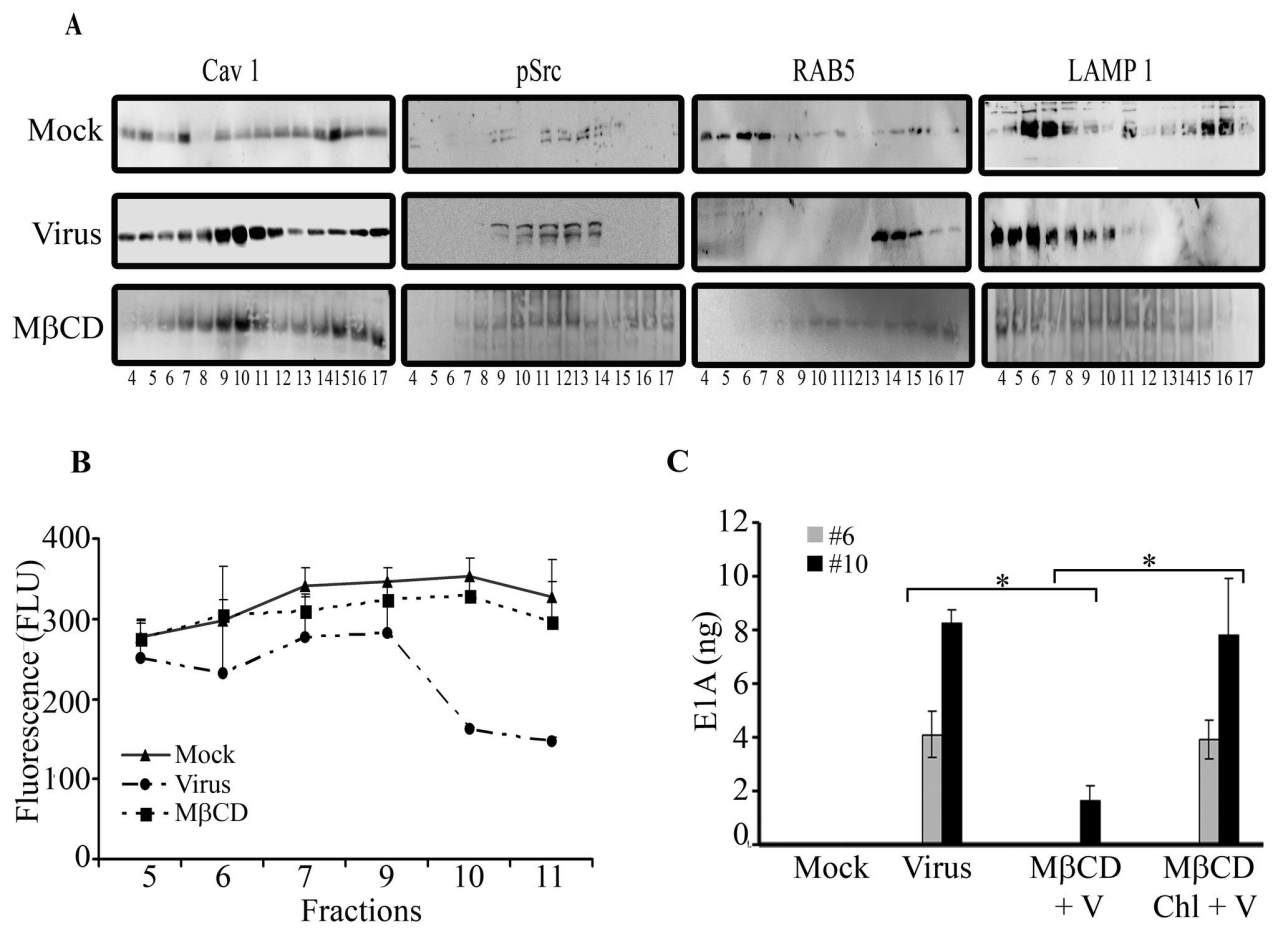
HAdV-D37 uses primarily caveolin-1 to enter corneal cells. **A**. Endosomal purification was performed on postnuclear extracts from corneal cells at 1 hr post infection in 62%, 35% and 25% sucrose solution. Fractions were immunoblotted for caveolin-1 (Cav 1), pSrc, RAB5, and LAMP1. **B**. Src kinase assay was performed on endosomal fraction #’s 5-11. In this assay, fluorescence is inversely proportional to kinase activity. As shown, fractions 10 and 11, which previously showed increased caveolin-1, also demonstrated greater Src kinase activity. **C**. Fractions that had maximum expression of LAMP1 (#6) and caveolin-1 (#10) were then subjected to real time PCR. For both fractions, the quantity of viral DNA was reduced by MβCD pretreatment (*p<.05). Cholesterol replenishment resulted in restoration of E1A expression (*p<.05). There was no difference in E1A expression between virus infected cells and those pretreated with MβCD but replenished with cholesterol and then virus infected. The data presented is representative of four experiments each run in duplicate.

Our results indicate that cholesterol depletion in corneal cells presents an impediment to entry of HAdV-D37 past the cell membrane. To investigate the type of endosome used in HAdV-D37 entry, flotation gradient fractionation of post nuclear supernatants was performed to separate different endosomal compartments [55]. Upon viral infection, fractions 4-6 maximally expressed LAMP1 indicating the presence of late endosomes [56], while fractions 9-11 were enriched with caveolin-1, indicating the presence of caveolae (Figure 3A). When the blots were stripped and reprobed, the caveolin-1 containing fractions were found to also contain pSrc. RAB5, a marker for early endosomes [57] was observed in fractions 14-16 upon viral infection, but was not evident in the fractions expressing pSrc. We confirmed Src activation in fractions 10 and 11 using a Src kinase assay in which substrate fluorescence and kinase activity are inversely proportional (Figure 3B). These results suggest that a pathway mediated by caveolin-1, and involving Src kinase, is an important route of HAdV entry into human corneal cells. MβCD pretreatment altered the fractionation of caveolin-1, and resulted in reduced identification of endosomal markers (Figure 3A). However, we cannot exclude alternative possible effects of MβCD on clathrin mediated mechanisms, as previously shown [58,59]. 

Real-time PCR was then performed to amplify HAdV-D37 genomic E1A nucleotide sequence [54] in order to determine the presence of viral DNA in different endosomal fractions. Upon infection of corneal cells, significantly more viral DNA was present in the fraction 10, containing caveolin-1and pSrc, than in fraction 6, which was abundant in LAMP1 (Figure 3C) (fraction 6 vs. fraction 10 in virus infected cells: p=.0027). MβCD pretreatment reduced viral DNA content in both endosomal fractions (p<.05, both comparisons). Viruses can often circumvent artificial barriers to cell entry by use of alternate entry pathways [50,60,61]. However, in our study, MβCD pretreatment reduced the presence of viral DNA in both the caveolin-1 containing fraction and in the LAMP1 containing fraction. We also examined for the presence of viral DNA in lipid raft fractions from MβCD treated cells when replenished with cholesterol, as compared to those treated with MβCD alone prior to viral infection. In lipid raft fractions from MβCD treated, cholesterol replenished cells, E1A content was equivalent to that from cells not treated with MβCD prior to infection (Figure 3C).

### Caveolin-1 and cholesterol are necessary for viral entry

**Figure 4:**
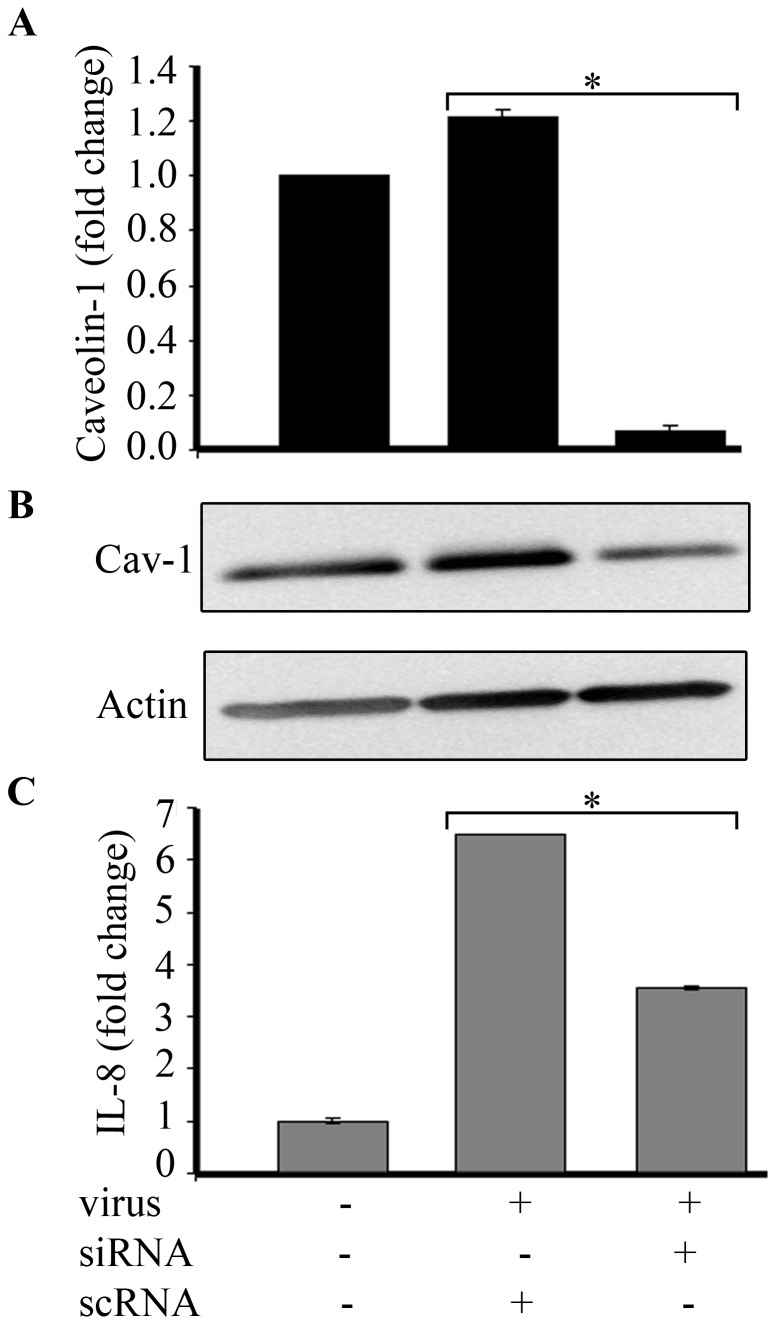
Caveolin-1 and cholesterol dependent viral entry. siRNA and scrambled (sc)RNA were generated and transfected into human corneal cells. **A**. Real-time RT-PCR for caveolin-1 mRNA confirmed successful reduction of caveolin-1 message (~95%) as compared to scRNA transfected cells (*p=.0002). **B**. Western blot showed reduced expression of caveolin-1 in caveolin-1 specific siRNA treated cells (right lane) as compared to untransfected and uninfected cells (left lane) or scRNA transfected, virus infected cells (middle lane) **C**. IL-8 mRNA expression after HAdV-D37 infection was reduced to almost 50% by caveolin-1 siRNA transfection, compared to scRNA (*p=.0001).

Caveolin-1 is a critical protein to caveolae [62], and upon reduction in caveolin-1 expression, cells lose the ability to form caveolae. We prepared a siRNA oligo against caveolin-1 that by real-time PCR reduced its expression in corneal cells to ~5% of normal (p=.0002, Figure 4A). Western blot analysis after siRNA transfection also showed reduction in caveolin-1 protein as compared to untransfected or control siRNA (Figure 4B). Specificity of the caveolin-1 siRNA was validated by showing expression of unrelated protein kinase C was not reduced compared to control (data not shown). However, HAdV-D37 infection of corneal cells after caveolin-1 knock down by siRNA reduced IL-8 expression by ~ 50% (p=.0001, Figure 4C). 

**Figure 5:**
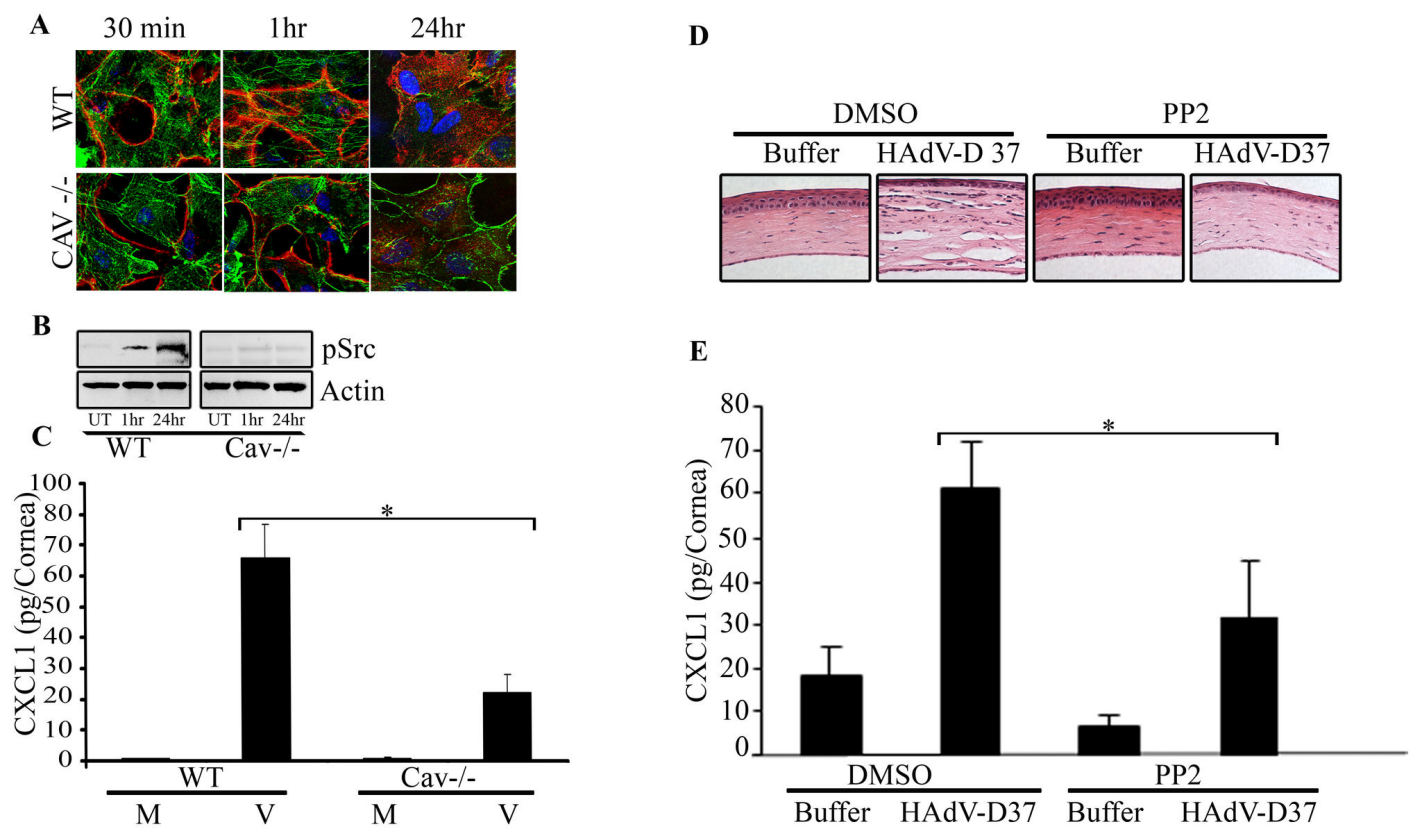
The role of caveolin-1 and Src kinase in HAdV entry and inflammation. **A**. C57BL/6J wild type (WT) and caveolin-1 -/- mice (on the C57BL/6J background) were injected intrastromally with Cy3-labeled (red) HAdV-D37 (10^5^ tissue culture infectious doses) or buffer control, and the corneas harvested for confocal microscopy. Phalloidin (green) co-staining was used to better visualize the cells. Virus entry was apparent by 1 hr post infection in WT mice. In caveolin-1 -/- mice, virus remained mostly membrane bound in the first hour post infection and internalization appeared reduced at 24 hr compared to WT mice. **B**. Western blot analysis of the cornea from mice infected with HAdV-D37 revealed increasing pSrc at 1 and 24 hr post infection, while expression was minimal in untouched (uninfected) corneas. Caveolin-1 -/- mice did not show increased pSrc even after 24 hr infection. Actin controls are shown in the bottom panel. **C**. ELISA for CXCL1 chemokine performed on WT and caveolin-1 -/- mice corneas at 24 hr post infection with HAdV-D37. Infected caveolin-1 -/- mice corneas showed approximately 60% less CXCL1 expression as compared to infected wild type corneas (*p=.0001). Mock infected mice corneas did not produce any detectable CXCL1. **D**. Histology of PP2 or DMSO (control) pretreated corneas at 4 days post infection with HAdV-D37 or buffer control shows a reduction of keratitis with chemical inhibition of Src kinase. **E**. CXCL1 expression by ELISA of HAdV-D37 infected or mock infected corneas at 16 hr post infection pretreated with the Src kinase inhibitor PP2 (10 μM) or DMSO control demonstrates a significant reduction in chemokine expression due to Src inhibition (*p<0.05).

Using the mouse adenovirus keratitis model [5,63,64], we then analyzed the role of caveolin-1 in viral infection. Cy3-labeled HAdV-D37 was injected into the corneal stroma of caveolin-1 -/- mice on a C57BL/6J background or wild type C57BL/6J controls. By confocal microscopy performed at 30 min, 1 hr, and 24 hr post infection, viral entry, but not viral attachment, appeared markedly reduced in caveolin-1 -/- as compared to wild type mice at these time points (Figure 5A). Western blot analysis of wild type and caveolin-1-/- mice corneas after infection revealed increased pSrc in wild type corneas at 1 and 24 hr post infection, compared to caveolin-1 -/- mice, suggesting that caveolin-1 is necessary for Src activation after infection (Fig, 5B). Similarly, upon viral infection, CXCL1 protein expression was reduced in caveolin-1 -/- mice as compared to wild type mice (p=.0001, Figure 5C). These results suggest that caveolin-1 is important to viral entry, kinase activation, and subsequent chemokine expression. 

Src kinase has been previously shown critical to both HAdV-D37 entry [35] and inflammatory consequences of infection of human corneal cells [35,52,65–67]. Data presented above further suggest that Src may play an important role in lipid raft mediated HAdV entry into corneal cells. Therefore, we applied the Src kinase inhibitor PP2 (10 μM) to the mouse adenovirus keratitis model. When injected 30 min prior to HAdV-D37 infection, PP2 reduced corneal inflammation as assessed by histology 4 days post infection (Figure 5D) and also reduced CXCL1 expression by ELISA at 16 hr post infection (p<.05, Figure 5E), while the PP2 solvent DMSO alone did not reduce inflammation or CXCL1 expression. Taken together, these data suggest HAdV-D37 enters corneal cells at least in part via caveolae, in an integrin dependent and Src kinase mediated pathway. 

### Ultrastructure of caveolin-associated viral entry

**Figure 6:**
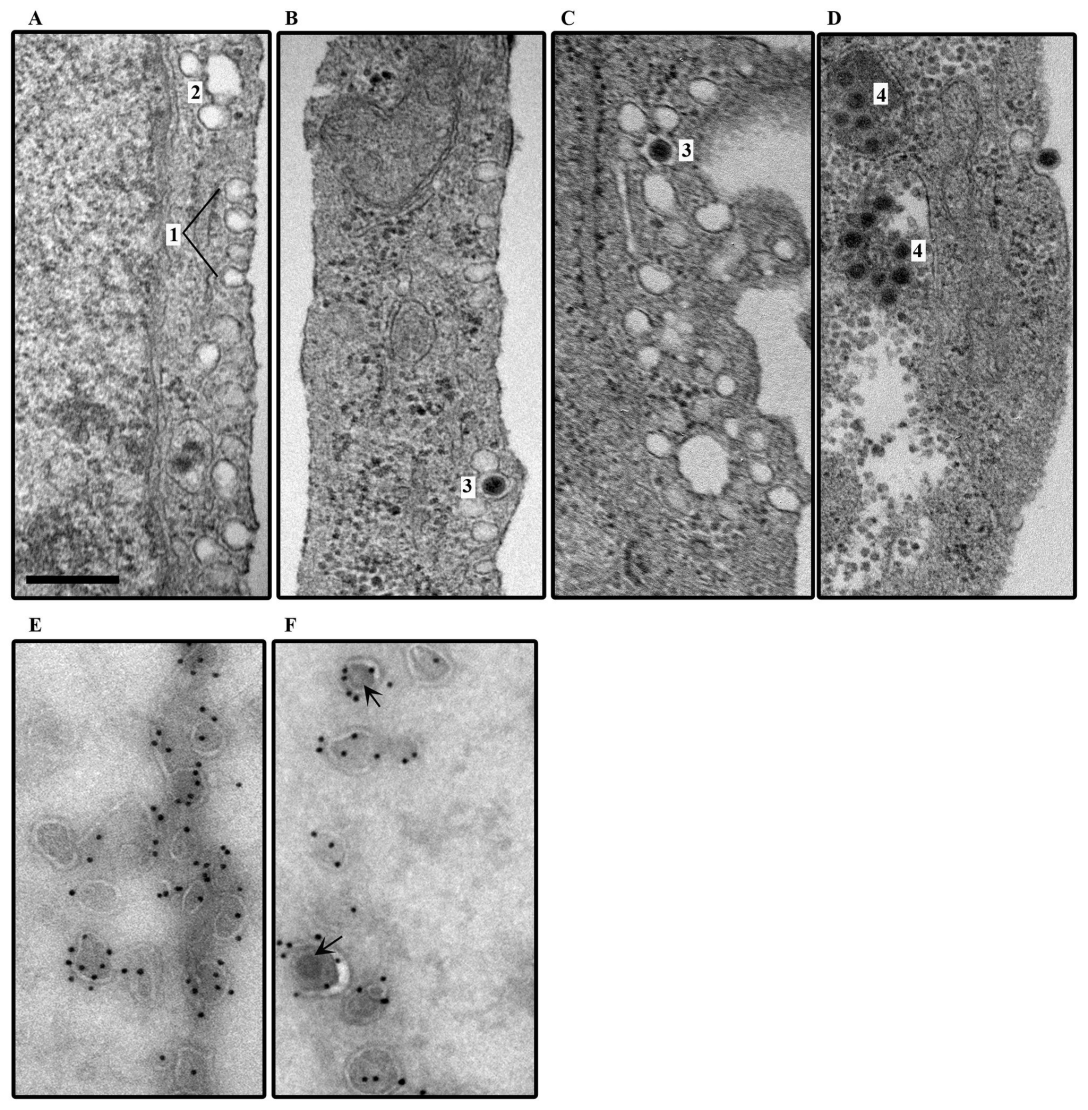
HAdV-37 enters human corneal fibroblasts through caveolin-1 containing, membrane associated vesicles. Human corneal fibroblasts were fixed in 2 % paraformaldehyde and the fixed, dehydrated cell pellet embedded and sectioned at 70-90 nm. Specimens were stained with uranyl acetate and Sato’s lead stain, and viewed by transmission electron microscopy. **A**. Photo-electron micrograph of cell membrane demonstrates multiple flask shaped vesicles resembling caveolae (1), and the formation of a cavesome-like structures (2). **B**-**D**. After 30 min of absorption at 4°C, and 30 min incubation at 37°C, virus can be seen within similar structures (3), and in some cases within what appear to be early or late fusions of caveolae to form caveosomes (4). Immunoelectron microscopy for caveolin-1 (**E**, **F**) was performed on cells infected as above, but treated with 2.3 M sucrose in PBS for 15 min, frozen, and sectioned at -120° C at 50-80 nm thickness. After immunostaining, protein-A nanogold was visualized by electron microscopy. (**E**) Uninfected cells stained for caveolin-1 demonstrate binding of 10 nm gold particles to flask-shaped vesicles. (**F**) After virus infection, virus (arrows) can be seen within vesicles also associated with gold particles. Scale bar = 500 nm.

Caveolae are submicroscopic, membrane associated vesicles found abundantly in some but not all mammalian cells. A *Caveola* has a characteristic flask or bulb-shaped appearance [68–70], without the obvious coat of clathrin pits[ 71]. Initially, SV40 was reported to use the caveolar pathway to enter cells [15,72], but was later observed to use a caveolae independent pathway [16,60,73]. Each caveolae contains approximately 140-150 caveolin-1 molecules [74]. To confirm caveolin associated viral entry into corneal fibroblasts, we performed ultrastructural studies with and without viral infection (Figure 6). Electron micrographs of uninfected fibroblasts demonstrate abundant membrane associated vesicles, visible as flask shaped invaginations of the cell membrane (Figure 6A). Within 1 hr after HAdV-D37 infection, viruses were seen in some of these *Caveola*-like structures (Figure 6B-D). In some tissue sections, it appeared that caveolae were fusing to form caveolar vesicles (caveosomes) as reported earlier [75]. Viruses also accumulated in these presumed caveosomes (Figure 6D). In order to confirm that the membrane associated vesicles seen ultrastructurally are indeed caveolae, we applied immuno-electron microscopy with primary antibodies against caveolin-1 or clathrin, and secondary antibody bound to protein-A nanogold. Membrane-associated vesicles in human corneal fibroblasts labeled well with antibody against caveolin-1 (Figure 6E). Within 30 min after viral adsorption, many caveolin-1 expressing vesicles were seen to contain virus (Figure 6F). The size of the caveolin-1 containing vesicles in corneal cells ranged from ~80-140nm in diameter (data not shown). Therefore some vesicles appeared able to accommodate the ~ 90 nm diameter adenovirus. Notably, clathrin staining vesicles in the same cells were much less abundant and no clathrin staining vesicles were seen to contain virus (data not shown). These results confirm that HAdV-D37 uptake into human corneal fibroblasts occurs in vesicles containing caveolin-1. 

## Discussion

Endocytosis is used by pathogens as a gateway to cellular entry. The specific pathway of entry seems to be host and pathogen specific [76]. The most well studied mechanism of receptor-mediated entry is clathrin-dependent endocytosis. Most previous reports suggest that HAdVs use clathrin mediated endocytosis for cellular entry [77,78], with macropinocytosis as an alternate pathway [77,79–81]. Caveolae are ubiquitous on the surface of cells, particularly in fibroblasts [82], and appear large enough to accommodate human adenoviruses [83–85], which typically measure ~ 90 nm in diameter. Recently, HAdV-C was shown to utilize lipid raft mediated, caveolar endocytosis in non fibroblast cell lines [50,61]. Because of our longstanding interest in pathogenesis of HAdVs in the human cornea, we sought to test viral entry in primary human corneal fibroblasts. We used two cell types, primary fibroblasts cultured directly from human donor corneas, and as control, A549 cells, a human alveolar epithelial cell line that was previously shown to support HAdV-1 virion production [86] and commonly used for the culture of HAdVs. Our results clearly demonstrate caveolin-1 associated entry into corneal cells by the highly cornea-tropic HAdV-D37. In contrast, HAdV-D37 entry into A549 cells was less robust and appeared to predominantly involve the LAMP1 pathway. As an additional control, we also tested the entry of HAdV-C2, a non corneal pathogen, but the degree of entry into human corneal cells was too low to permit quantitative analysis. Caveolae mediated entry of another non-enveloped DNA virus, SV40, is controversial [16,60,73], and in the absence of caveolin-1, SV40 still successfully enters cells [60], suggesting redundant and alternative mechanisms. However, in corneal stromal cells, HAdV-D37 appears to rely largely on lipid raft mediated, caveolin-1 associated endocytosis. Cholera toxin B subunit (CTXB) has been shown to bind glycosphingolipid ligand, GM1, and has been used to identify distinct, lipid raft endocytic pathways [87]. In our study, CTXB co-localized with HAdV-D37 in corneal cells during entry. We show the presence of viral DNA predominantly in caveolin-1 associated membrane fractions. It is important to acknowledge that clathrin-mediated endocytosis is also sensitive to acute depletion of cholesterol [58,88] and that raft recruitment has been shown to precede clathrin-dependent endocytosis for anthrax toxin, EGFR, and BCR, respectively [89–91]. In our study, cholesterol depletion also reduced viral DNA in LAMP1 rich fractions in corneal cells, indicating a similar redundancy in cholesterol function. When cholesterol was replenished in cells treated with MβCD, there was a gain in entry as determined by confocal microscopy and viral DNA expression. We also previously showed the critical role of Src in HAdV infection [35,52,66,67,92]. The observation of increased Src kinase activity specifically in caveolin-1 enriched fractions in infected cells strongly suggests a role for caveosomal signaling upon viral infection. At 1 hr post infection, PP2, a chemical inhibitor of Src kinases, partially reduced viral entry, whereas both MβCD and RGD more completely blocked viral entry. Using a mouse keratitis model [63], caveolin-1-/- mice showed reduced entry of virus as compared to wild type mice and showed reduced levels of both pSrc and CXCL1 expression complimenting our *in vitro* data. Taken together, these findings suggest a role for Src kinase in the caveosome during viral entry, with downstream effects on chemokine expression by infected cells. 

Viruses even within the same virus family can utilize different endocytic pathways, and there may be extensive cross talk between pathways. For example, within the polyomaviruses, JC virus enters cells via clathrin mediated endocytosis [93] and is later sorted into caveosomes [94], while BK virus uses pH dependent caveolar endocytosis [95]. Other polyomaviruses enter cells using uncoated vesicles independently of both caveolin and clathrin [96]. These studies do not account for potential differences in cell types used to determine viral entry. Our ultrastructural studies confirm the association between HAdV-D37 with caveolin-1 in corneal fibroblasts during viral entry, but not clathrin in the same cells. Our data also suggests that HAdV-D37 may utilize different entry pathways in different cell types. Time course studies with a panel of cell types relevant to corneal infection will be necessary to determine the range of entry mechanisms utilized by HAdV-D37, and to better understand the infectious and inflammatory outcome of different entry pathways. HAdV-D37 replicates readily in human corneal fibroblasts [53], and in our hands, knockdown of caveolin-1 reduced chemokine expression, suggesting that caveolin contributes to both productive infection and corneal inflammation. However, our data may be relevant only to the earliest stages of infection, and does not address possible sorting of virus later in infection to different endosomal compartments.

In summary, we have shown that HAdV-D37 enters corneal stromal cells via a caveolin-1 associated, lipid raft specific pathway, inducing caveosomal signaling and expression of pro-inflammatory mediators. Lipid raft mediated endocytosis may prove a useful target for antiviral therapy in corneal infection.

## Experimental Methods

### Ethics Statement

The animals involved in this study were procured, maintained, and used in accordance with the Laboratory Animal Welfare Act of 1966, as amended and NIH 80-23, Guide for the Care and Use of Laboratory Animals, National Research Council, and upon approval from the Animal Care Committee at the Mass. Eye and Ear Infirmary. The derivation of corneal cells from deceased human donors was approved by the Massachusetts Eye and Ear Infirmary Human Studies Committee, and conformed to the tenets of the Declaration of Helsinki; written informed consent for the use of the corneas from the deceased donors or their families was deemed unnecessary by the Human Studies Committee, because the corneal tissues arrived without identifying data, and were tissues that were found by the eye banks to be unsuitable for corneal transplantation and would have been discarded if not used in research.

### Cells and virus

Primary human corneal fibroblasts were derived from human donor corneas as previously described [97]. Cells from multiple deceased donors from multiple eye banks were pooled and the cell monolayers used at passage two or three. The A549 cells and HAdVs used in this study were purchased from American Type Culture Collection (ATCC, Manassas, VA). Virus purification was performed by cesium chloride gradient. Cy3-labeled virus was prepared as previously described [98].

### Mice

Eight to 12-week-old wild type C57BL/6J and caveolin-1 -/- mice were obtained from Jackson Laboratories (Bar Harbor, ME). For experimental infection, female mice were anesthetized by intramuscular injection of ketamine (85 mg/kg) and xylazine (14 mg/kg). Anesthetic drops (0.5% proparacaine hydrochloride, Alcon, Fort Worth, TX) were applied topically to the right cornea, followed by injection with 1 μl PP2 (10 μM), DMSO carrier, and then HAdV-37 after 1 hour. One μl of virus (10^5^ tissue culture infective dose (TCID)), or virus-free dialysis buffer was injected into the central corneal stroma with a glass micropipette needle fitted with a gas-powered microinjection system (MDI, South Plainfield, NJ) under an ophthalmic surgical microscope (Carl Zeiss Meditec, Inc., Thornwood, NY). At 4 days post infection, mice were euthanized using CO_2_ inhalation prior to removal of corneas for histology. 

### Confocal Microscopy

Cells grown on slide chambers (Nunc, Rochester, NY) were treated with MβCD (5mM; Sigma, St. Louis, MO), RGD 15-mer (50mM, GenScript, Piscataway, NJ ), KGE 15-mer (50mM, GenScript, Piscataway, NJ) or PP2 (10 μM, Calbiochem, La Jolla, CA) for 1 hr, and then infected with Cy3-labeled HAdV for 30 min and 1 hr. Cells were partially fixed in 0.05% paraformaldehyde for 10 min, washed in PBS containing 2% FBS, and permeabilized in solution containing 0.1% Triton X-100 for 5 min. After 30 min blocking in 3% FBS-PBS, the cells were incubated in 5 μg/ml of Alexa Fluor 488 phalloidin (Invitrogen, Eugenia, OR) for 30 min at room temperature, washed three times in 1x PBS containing 2% FBS. Cells were then fixed in 2% paraformaldehyde, and mounted using Vectashield (Vector labs, Burlingame, CA) mounting medium containing DAPI. Images were taken in a Leica SP5 confocal microscope using a 63x glycerol immersion objective. Mouse corneas were harvested at various time points after HAdV-D37 injection and stained with Alexa Fluor 488 phalloidin, (Invitrogen), fixed, and mounted using Vectashield. For co-localization studies after infection, cells were fixed and stained with antibodies to caveolin-1, (Santa Cruz Biotechnology, CA) LAMP1, (Santa Cruz Biotechnology), or 488-CTXB, (Invitrogen, OR). Individual confocal image stacks were loaded to measure co-localization pixels by Amira 5.2.2 (Amira, Burlington, MA) from cells stained green (caveolin-1 or LAMP1) and red (Cy3-labeled HAdV-D37). Three frames each from 3 separate experiments were chosen in masked fashion and, used to generate mean and standard deviation.

### Non-detergent isolation of lipid rafts

Cells were treated with MβCD, washed thoroughly, and infected with purified HAdV at a multiplicity of infection (MOI) of 10 for 30 min or mock treated with dialysis buffer. After infection, LR fractions were isolated using detergent free methods. Briefly, cells were washed once in ice cold PBS, suspended in 500mM Na_2_CO_3_, and lysed by 20 strokes in a prechilled dounce homogenizer. Further disruption of cell membranes were achieved by passing the lysate through a 23 gauge needle 5 times, followed by 3 cycles of sonication for 15 s. The subsequent lysate was mixed with an equal volume of 90% sucrose-MBS (25mM MES{2-(N-morpholinoethanesulfonic acid) plus 0.15M NaCl [pH 6.5]), overlaid on an equivalent volume of 35% sucrose-MBS- Na_2_CO_3,_ and centrifuged for 16-18 hr at 4°C. 500μl fractions were collected and the protein concentrations measured. The lysates were immunoblotted with anti-pSrc (Cell Signaling, Danvers, MA) to identify phosphorylated Src in LR along with the LR marker caveolin-1. 

### Endosome isolation

Flotation gradient fractionation of post nuclear supernatants was performed with 62%,(2.351M) 35%, (1.177M) and 25%, (0.806M) sucrose gradients [55] and 100 μl fractions collected. One hr post HAdV infection after pretreatment with MβCD (5mM), untreated controls and mock infected cells were collected and post nuclear supernatants were prepared by using NucBuster (Novagen, Germany). Post nuclear supernatants were then mixed with an equal amount of 62% sucrose, layered over with 1.5 volume of 35% sucrose and 1 volume of 25% sucrose, and centrifuged for 1 hr at 149,000 x *g* at 4°C. A series of 100 μl fractions were collected from the surface and subjected to western blot analysis for caveolin-1, phosphorylated Src, RAB5, and LAMP1 (Santa Cruz Biotechnology), and real-time PCR for the HAdV-D37 E1A gene in order to determine the presence of viral DNA in the endosomes.

### siRNA construction and transfection

The siRNA for caveolin-1 and its control scrambled siRNA (Table 1) were constructed using SciTools from Integrated DNA Technology (IDT, Coralville, IA). One nm was transfected using Lipofectamine RNAiMAX (Invitrogen, Carlsbad, CA) following the manufacturer’s instructions. At 48 hr post transfection, cells were infected with HAdV for 2 hr, and then analyzed for caveolin-1 and IL-8 message using real-time RT-PCR (Table 1). For Western blot analysis, 20 μg of protein was separated in a 4-20% gradient gel (Invitrogen) and immunoblotted with antibodies against caveolin-1 and actin (Thermo Scientific, Pittsburgh, PA), the latter as an internal control.

**Table 1:** Nucleotide sequences for siRNA and PCR.

**siRNA**	Sense Sequences	Antisense Sequences
Caveolin-1 siRNA	rCrCrA rUrCrU rArCrG rUrCrC rArCrA rCrCrG rUrCrU rGrUG A	rUrCrA rCrArG rArCrG rGrUrG rUrGrG rArCrG rUrArG rArUrG rGrArA
Caveolin-1 scRNA	rGrGrU rArCrA rGrGrC rGrArU rGrArA rUrArG rGrUrC rGrGrC rU	rArGrC rCrGrA rCrCrU rArUrU rCrArU rCrGrC rCrUrG rUrArC rC
**PCR**	Forward Primers	Reverse Primers
E1A	TTCCTCCCAGCGATTCAGAG	GGGCACCTCAGGATTGTCC
Caveolin-1	GGGCATTTACTTCGCCATTC	ACGGTGTGGACGTAGATGGA
IL-8	TGCAGCTCTGTGTGAAGGTG	ATTTCTGTGTTGGCGCAGTG
GAPDH	TGGGCTACACTGAGCACCAG	ACCACCCTGTTGCTGTAGCC

### Real-time PCR

To quantify the presence of viral DNA in endosomal compartments, the E1A gene was amplified from endosomal fractions after sucrose gradient with primers (IDT, Table 1) designed based on the HAdV-D37 genomic E1A nucleotide sequence [54]. Real-time PCR was performed with Fast SYBR Green master mix (Applied Biosystems, Foster City, CA) with the following conditions, 40 cycles at 95°C (10 s), 55°C (20 s), and a final extension at 72°C for 10 min. To determine mRNA expression for caveolin-1 and IL-8, total RNA was extracted from caveolin-1 or scrambled siRNA transfected, HAdV-D37 infected cells, at 2 hr post-infection using TRIzol (Invitrogen) according to the manufacturer’s instructions. Total RNA was then treated with DNase I (1 unit) (New England BioLabs, Ipswich, MA) at 37°C for 1hr. Two μg of the DNase treated RNA was subjected to reverse transcription with M-MLV reverse transcriptase (Promega, Madison, WI) and oligo dT_15_ primers (IDT). Primers for caveolin-1 and IL-8 were designed using Primer3 plus software (http://www.bioinformatics.nl/cgi-bin/primer3plus/primer3plus.cgi), and synthesized by IDT (Table 1). The cDNAs were diluted by 1:10 and 1 µl was used for qRT-PCR using Fast SYBR Green master mix under the conditions: 40 cycles oat 95°C (30 s), 60°C (1 min), 72°C (30 s), and a final extension at 72°C for 10 min. A non-template control and endogenous control (human GAPDH) were measured for the relative quantification. The related expression levels were calculated by the 2^-ΔΔCT^ method against the untransfected/uninfected controls [99].

### Src kinase assay

Src activity assays were performed by using ProFluor® Src-Family Kinase Assay (Promega, Madison, WI). One microgram of each endosomal fractions (peptide substrate) and 25 μl of 100 μM ATP was added to the reaction buffer and incubated for 60 min at room temperature, followed by the addition of protease reagent for another 60 min at room temperature. The reaction was then stopped by addition of stabilizer reagent. When there is no kinase activity, the peptide substrate remains nonphosphorylated, and the protease will remove all amino acids from the peptide substrate and liberate the highly fluorescent rhodamine 110 (R110). In the presence of active kinase, the peptide substrate will be phosphorylated, effectively blocking the protease activity and resulting in low fluorescence of the Src-Family Kinase R110 substrate. The resulting fluorescence is read at excitation wavelength 485 and emission wavelength of 530 nm.

### ELISA

Mouse corneas were removed at 16 hr post infection (n = 3/group). Corneas were then homogenized in 400 µL of PBS with 1 mM phenylmethylsulfonyl fluoride (PMSF), 1 µg/mL aprotinin, and 10 µg/mL leupeptin (Sigma-Aldrich, St. Louis, MO). The lysates were centrifuged at 10,000 x *g* for 10 minutes at 4°C, and the supernatants were used for ELISA. Mouse CXCL1 (KC) (R&D Systems, Minneapolis, MN) protein detection was performed with commercially available sandwich ELISA kits with capture and detection antibodies, according to the manufacturer’s instructions. Each sample and standard was analyzed in duplicate. The plates were read on a microplate reader (Molecular Devices, Sunnyvale, CA) and analyzed (SOFTmax; Molecular Devices). 

### Histopathology

Upon euthanasia at 4 d post infection, mouse corneas were removed surgically, rinsed in PBS, and fixed with 10% neutral buffered formalin for 24 hr at room temperature. After paraffin embedding, whole eyes were cut into 5-μm-thick sections, mounted on positively charged slides and air dried overnight. After deparaffinization and rehydration, slides were stained with hematoxylin and eosin for histology, coverslipped, and photographed (Axiovert 135; Carl Zeiss Meditec, Inc.), using a 40× objective. 

### Electron Microscopy

Human corneal fibroblasts plated on 10 cm dishes and grown to 95% confluence were infected with cesium chloride purified HAdV-D37 virus at a MOI of 10, and incubated at 30 min at 4° C, followed by incubation at 37° C for 30 min. The cells were then washed with PBS, and fixed in 2 % paraformaldehyde containing 2.5 % gluteraldehyde, 0.1 M cacodylate and 2.5 mM CaCl_2_ for 1 hr, and harvested in 2 % agarose. The cell pellet was fixed for 1.5 hr in 2 % aqueous OsO_4_, and dehydrated. Following dehydration, the cell pellet was embedded in epon and sectioned at 70-90 nm. The specimens were stained with saturated aqueous uranyl acetate, and Sato’s lead stain. Photomicrographs were taken on a Philips CM-10 electron microscope (Koninklijke Philips Electronics N.V., Amsterdam, Netherlands) operating at 80 kv and fitted with a CCD camera.

### Immunogold Electron Microscopy

For immunostaining, cells were adsorbed with purified HAdV-D37 or mock infected with buffer for 30 min at 4° C, followed by incubation at 37° C for 30 min, washed in PBS, fixed in 4% paraformaldehyde for 1 hr, and harvested. Cells were treated with 2.3 M sucrose in PBS for 15 min, frozen and sectioned at -120° C at 50-80 nm thickness, and transferred to formvar-carbon coated copper grids. Grids were blocked with 1% BSA for 10 min and incubated for 30 min with 5 µl of rabbit polyclonal caveolin-1 antibody, (BD Biosciences, San Jose, CA) at 25 µg/ml in 1% BSA, followed by wash in PBS, and incubation with protein-A nanogold (Cell Microscopy Center, Department of Cell Biology, University Medical Center, Utrecht, The Netherlands) [100] with a particle size of 10 nm, for 20 min. Alternate sections were stained with mouse IgG1 raised against rat clathrin heavy chain (BD Biosciences) followed by rabbit antiserum to mouse IgG (ICN Biomedicals, Irvine, CA) at 1:100 dilution, followed by protein-A nanogold with a particle size of 5 nm. Protein-A nanogold was incubated for 20 min followed by washing in PBS, and a final wash with double distilled water. Grids were then stained in 0.3% uranyl acetate in 2% methyl cellulose for 10 minutes and photomicrographed in the electron microscope. 

### Statistical analysis

Comparisons of means were performed with ANOVA with Sheffe’s multiple comparison test, or where appropriate with Students *t* test. p<.05 was considered statistically significant.
